# Steerable needles for radio-frequency ablation in cirrhotic livers

**DOI:** 10.1038/s41598-020-77869-3

**Published:** 2021-01-11

**Authors:** Nick J. van de Berg, Frédérique C. Meeuwsen, Michail Doukas, Gernot Kronreif, Adriaan Moelker, John J. van den Dobbelsteen

**Affiliations:** 1 Dept. of Biomechanical Engineering, Delft University of Technology, Mekelweg 2, 2628 CD Delft, The Netherlands; 2 Dept. of Radiology and Nuclear Medicine, Erasmus MC, University Medical Center, Rotterdam, The Netherlands; 3 Dept. of Pathology, Erasmus MC, University Medical Center, Rotterdam, The Netherlands; 4 Austrian Center for Medical Innovation and Technology, Wiener Neustadt, Austria

**Keywords:** Biomedical engineering, Oncology

## Abstract

Accurate needle placement in deep-seated liver tumours can be difficult. In this work, we disclose two new manually controlled steerable needles for 17G radio-frequency ablation probe placement. The needles contain stylets with embedded compliant joints for active tip articulations, and concentric tubes for (curved-path) guidance. Needle steering was evaluated sequentially by intended users and in intended-use tissue types. Six interventional radiologists evaluated the needle in repeated ultrasound-guided steering tasks in liver-mimicking phantoms. Targets were located at a 100 mm depth and 20 mm lateral offset from the initial insertion line. The resulting mean absolute tip placement error was 1.0 ± 1.0 mm. Subsequently, steering-induced tissue damage was evaluated in fresh cirrhotic human liver explants. The surface area of puncture holes was estimated in scanned histology slides, using a connected-components analysis. The mean surface area was 0.26 ± 0.16 mm^2^ after steering with a median radius of curvature of 0.7 × 10^3^ mm, versus 0.35 ± 0.15 mm^2^ after straight-path insertions with the steerable needle and 0.15 ± 0.09 mm^2^ after straight-path RFA probe insertions. The steering mechanisms proposed enable clinically relevant path corrections for 17G needles. Radiologists were quickly adept in curved-path RFA probe placement and the evaluation of histological tissue damage demonstrated a potentially safe use during liver interventions.

## Introduction

### Clinical background

Liver cancer, including hepatocellular carcinoma (HCC), accounts for an annual 841,000 new cases and 782,000 cancer-related deaths worldwide^1^. It is one of the few cancers with a still increasing incidence and death rate, contrasting the overall drop in cancer death rates by 23% over the last two decades^2^. Surgical resection is the treatment of choice for HCC, although resection criteria in accordance with the American Association for the Study of Liver Diseases (AASLD) and European association for the Study of the Liver (EASL) are met in only 10% of patients^3^. In patients with small HCC and metastases, radiofrequency ablation (RFA) is a viable alternative as primary treatment for reproducible local tumour control with minimal morbidity^5^. Ablation is advised for HCC stage 0 and A in the Barcelona Clinic Liver Cancer (BCLC) staging system^6^. For nodules ≤ 30 mm, RFA resulted in similar life expectancy and quality-adjusted life expectancy compared to resection, at lower costs^7^.

Optimal target coverage and minimal damage to surrounding healthy tissue requires an accurate RFA probe placement. However, undesired deflections of needles in the liver increase with target depth^8^ and complicate needle placement tasks^4^. Typical causes of deflection are respiratory motions and unbalanced forces acting on the needle when traversing heterogeneous structures. In addition, not all targets are equally well reachable to start with, e.g. when situated in the deeper liver segments I, VII and VIII. In a questionnaire among 125 experienced radiologists, 90% of respondents indicated that needles in radiology should be improved in terms of manipulability^4^. Respondents considered liver ablation a top application for technological research and development.

### Related work and contribution

To improve manipulability and enable path rectifications, needle steering mechanisms have been developed^9–12^. A hand-held steerable needle with battery-powered actuation of a pre-curved stylet protruding from a rigid cannula was proposed by Okazawa et al.^13^. Tip-articulated needles were studied by van de Berg et al.^14^. Burdette et al.^15^ were the first to integrate an acoustic ablator within a needle steering device. Subsequently, Gerboni et al*.*^16^ evaluated radii of curvature during distributed ablations in porcine liver tissue. To date, few studies have been performed in human tissue. A new robotic control method for a 22G bevel-tipped biopsy needle was evaluated in fresh-frozen human lungs by Shahriari et al.^17^. Finally, Kriegshauser et al.^18^ recently published initial clinical outcomes with a commercially available steerable needle used for neurolysis injection (21G Morrison Steerable Needle, AprioMed). Needle steering was beneficial to avoid traversing organs at risk in 6/13 (46%) of interventions.

The goal of this study is to disclose two new, manually controlled steerable needles, describe their steering mechanism and demonstrate their functionality. In extension of the work above, the mechanism proposed is suitable for small (≥ 20G) and large (14–19G) diameter needles, i.e. using the needle size definitions of Cholongitas et al.^19^. Research on large-diameter needle steering is scarce, as these needles are generally stiffer, which interferes with steerability. Yet, their clinical applicability is high, e.g. many biopsy and ablation needles are available in sizes of 14–17G. The proposed mechanism therefore enables steering for a wider range of clinical tasks. The design uses a novel compliant mechanism without laterally protruding recesses, enabling tip articulations while maximally preserving flexure joint rigidity, e.g. needed for controlled interactions in tough and heterogeneous cirrhotic livers.

Needle steering functionality is evaluated in ultrasound-guided insertions performed by six interventional radiologists (intended users) in polyvinyl alcohol (PVA) phantoms. In addition, tissue damage resulting from load distributions exerted on tissue, necessary to form and maintain curved needle paths, is evaluated in fresh cirrhotic human liver explants (intended-use tissue types). To our knowledge, this is the first study in which actively steered needles are evaluated in human livers.

## Results

### Needle designs

**Figure 1:**
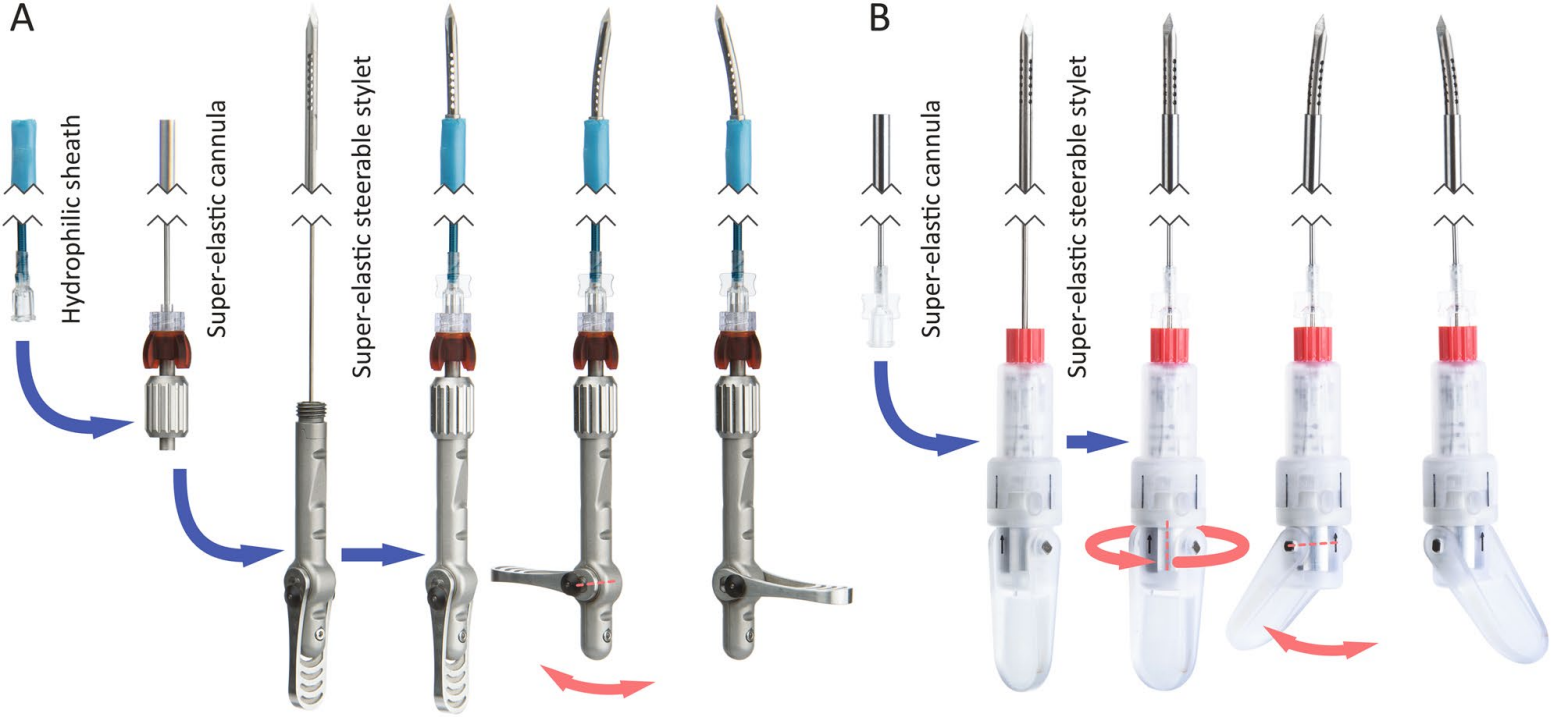
Modular designs of v1 planar (**A**) and v2 omnidirectional (**B**) steerable needles, with hydrophilic sheaths and nitinol stylets and cannulas. Compliant joints enable tip articulations of approximately 11°. Assembly steps are shown with blue arrows, and degrees of freedom in actuation with red arrows.

Manufactured needles are shown in Fig. 1 and relevant mechanical and functional characteristics are detailed in “Methods” section. Stylets and inner tubes are retractable and can be decoupled using standard Luer locks, while outer cannulas or sheaths serve to introduce commercially available needles. All tubes and transmission components are modular and interchangeable to accommodate different commercial needles and steering responses. The disclosed designs aim at placement of 17 G × 200 mm RFA probes.

Two needles were designed and manufactured that enable tip articulations with angles up to 11° in a planar (needle v1) and omnidirectional (needle v2) manner. The two iterations are similar in their working mechanism. The needle tip contains a compliant structures with internal recesses that balance flexibility and rigidity demands, and the device handle uses a lever to manually control the configuration at the tip. Needle v1 has an additional hydrophilic outer sheath, which increases its diameter to 2.60 mm, versus 1.80 mm for the cannula of needle v2.

### Evaluation part I: RFA probe placement

**Figure 2:**
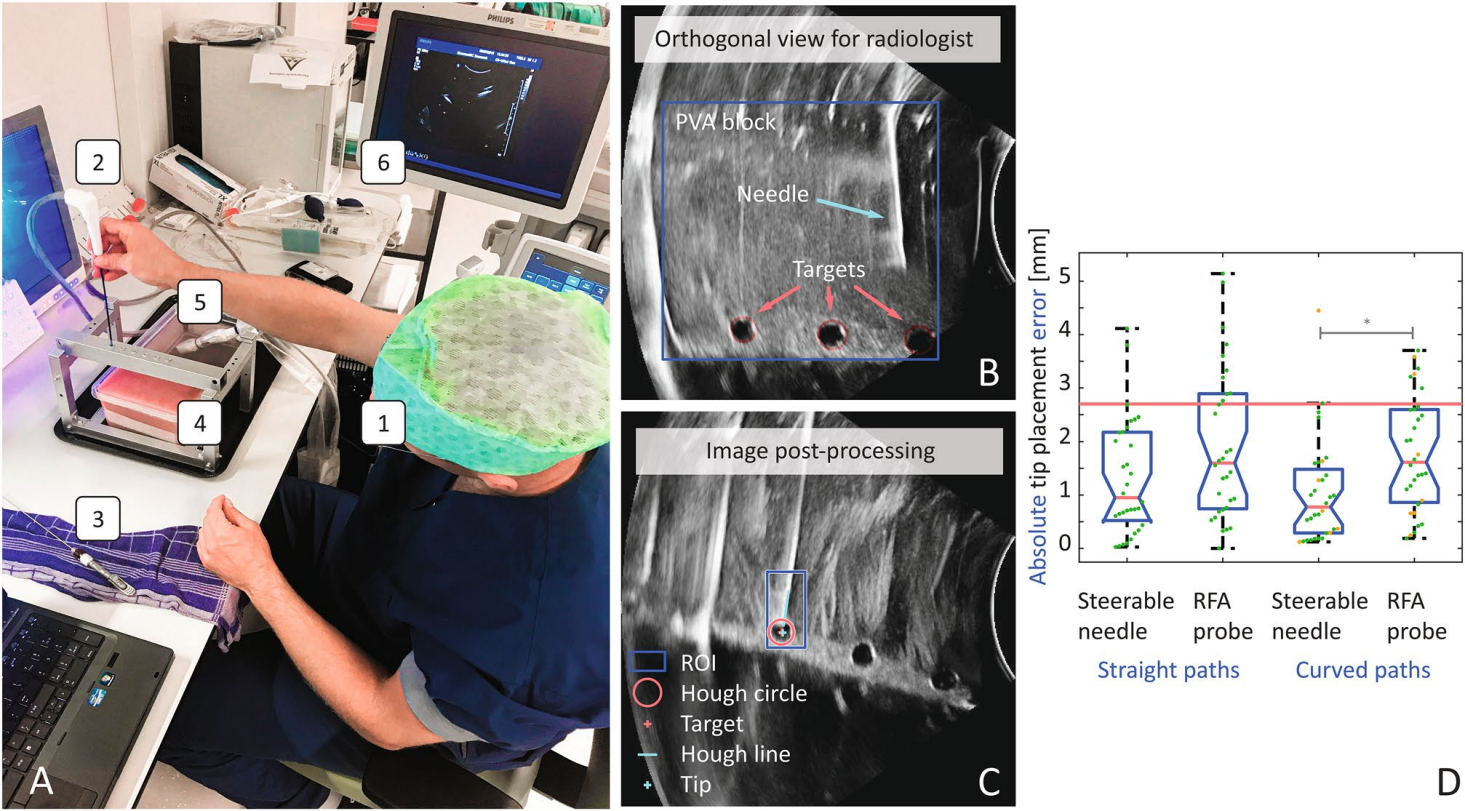
Image of the experimental platform (**A**), showing the interventional radiologist (1), RFA probe (2), steerable needle v1 (3), and PVA phantom (4), with a trocar bridge to control the insertion location, needle alignment and steering task. The ultrasound probe (5) is manually controlled to visualise insertions on-screen (6). Typically, radiologists pressed the probe to the side of the PVA specimens, yielding an orthogonal view (**B**). Ultrasound images were stored and processed, using a region of interest (ROI) and Hough transforms to estimate target, needle and tip locations (**C**). Tip placement errors for straight and curved paths are shown by boxplots and individual data points (**D**). The asterisk indicates a Wilcoxon rank sum test difference (α = 0.05). Errors after manual path correction steps are visualised in orange. The red line indicates an acceptance threshold error of 2.7 mm, which was the mean position error accepted by a group of 125 interventional radiologist^4^.

The research aim was to evaluate ultrasound-guided needle (v1) placement by interventional radiologists (Fig. 2A) along straight paths and curved paths, in which the target was located at a depth of 100 mm, with or without a lateral offset of 20 mm (Fig. 2B). After placement, the outer sheath was unlocked and the steerable needle was removed, leaving a working channel for RFA probe insertion (17 G × 200 mm Amica RFA probe, HS Hospital Service S.P.A., Italy). The steerable needle placement error (Fig. 2C) and times required for needle placement and instrument switching were registered. In addition, the effect of the instrument switch on the position error was registered, as this error was expected to change due to differences in flexural rigidity of the steerable needle and RFA probe.

Mean and standard deviation (SD) of times required to place needles and switch instruments (remove stylet and insert RFA probe) were 16 ± 8 s and 24 ± 7 s for straight paths. In case of curved paths, the same steps took 22 ± 17 s and 22 ± 9 s. Manual path corrections using the same tissue entry point were allowed and used for seven insertions (shown by orange datapoints in Fig. 2D), requiring two (n = 3), three (n = 3) or four (n = 1) tries to reach the target. This only occurred for curved needle paths.

Absolute tip placement errors are shown in boxplots in Fig. 2D. They were evaluated using a Kruskal–Wallis test and Wilcoxon rank sum tests (α = 0.05). The mean and SD of steerable needle placement errors along straight and curved paths were 1.3 ± 1.1 mm and 1.0 ± 1.0 mm, respectively. After the instrument switch, the RFA probe position errors were 1.9 ± 1.4 mm and 1.8 ± 1.1 mm, respectively. The Kruskal–Wallis test indicated a difference between groups (p = 0.005). The Wilcoxon rank sum tests located a difference in the curved-path placement of steerable needles and RFA probes (p = 0.002), i.e. caused by switching of instruments in the curved-path cases. Differences were not seen for straight path insertions (p = 0.07) or for curved and straight path placement using the steerable needle (p = 0.33).

### Evaluation part II: tissue damage after needle steering

**Figure 3:**
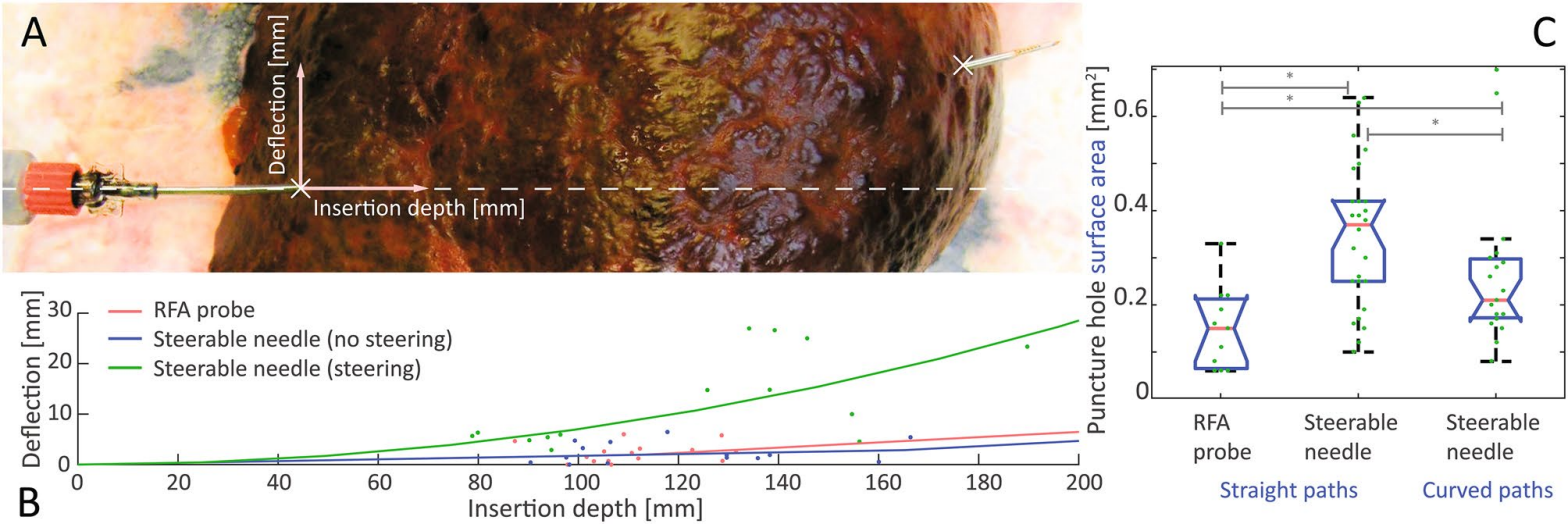
Illustration of insertion depth and needle deflection (**A**), and summary of deflection data (**B**) for punctures in fresh, cirrhotic liver explants (**A**), using the RFA probe and steerable needle v2, along straight and curved paths. Also shown are summary boxplots and individual data points (**C**) of histological tissue damage (mm^2^) after the insertions. The asterisks indicate Wilcoxon rank sum test differences (α = 0.05).

The aim of the second part of the functional evaluation was to compare cross-sectional areas of puncture holes after straight-path and curved-path insertion, with the steerable needle (v2) and RFA probe (Fig. 3A). This metric served to quantify damage caused by steering forces exerted on tissue. It should be noted that the RFA probe is smaller (cross-sectional area of 1.96 mm^2^) and fits within the concentric tube of the v2 steerable needle (2.54 mm^2^). A comparable analysis of cross-sectional areas was used to demonstrate that tissue damage can be reduced in duty-cycling based steering of bevel-tipped needles by using bidirectional rotations, i.e. avoiding wind-up of tissue^20^.

Needle exit point coordinates and path curvatures for the insertions in liver explants are shown in Fig. 3B. The fits illustrate the median constant radius of curvature arc for each experimental condition. Deflections measured 2.3 ± 1.9 mm for the RFA probe (mean depth of 110.9 mm), 2.4 ± 2.0 mm for the steerable needle for straight paths (mean depth of 119.7 mm), and 12.8 ± 8.9 mm for the steerable needle for curved paths (mean depth of 122.7 mm). The median radius of curvature of paths for these three conditions was 3.2 × 10^3^, 4.7 × 10^3^, and 0.7 × 10^3^ mm, respectively.

**Figure 4:**
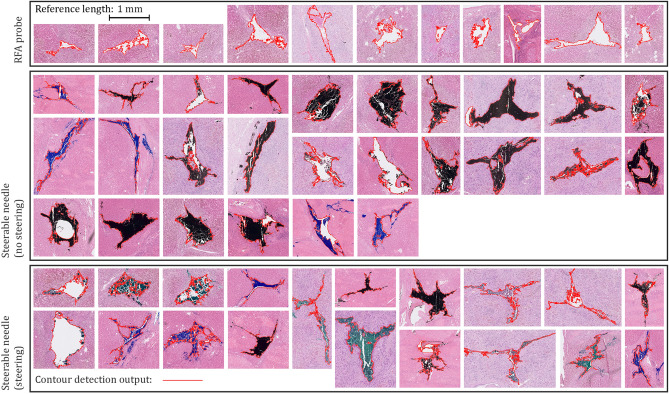
Close-ups of (inked) tissue damage sites in the histology slides. The results of a CCA-based automatic contouring step are shown as red lines.

Figures 3C and 4 show the collection of needle puncture holes for the RFA probe (n = 11) and steerable needle in insertions without (n = 26) and with steering (n = 19). The mean and SD of cross-sectional puncture hole areas were 0.15 ± 0.09 mm^2^, 0.35 ± 0.15 mm^2^ and 0.26 ± 0.16 mm^2^, respectively. The Kruskal–Wallis test indicated that these groups differ (p < 0.001). The Wilcoxon rank sum tests located differences in surface areas between RFA probes and steerable needles following a straight (p < 0.001) or curved path (p = 0.02), and between steerable needles following straight and curved paths (p = 0.05).

## Discussion

This research presents two new steerable needle designs and demonstrates: (1) their usability for interventional radiologists to correct paths in PVA phantom tissue, and (2) the limited resultant tissue damage while steering in fresh human liver explants. The needles enable bidirectional (v1) and omnidirectional (v2) tip articulations, resulting in curved trajectories during insertion. They allow placement of open working channels with modularly adjustable dimensions, via interchangeable stylets, cannulas and transmission components. Working channels are easily decoupled via Luer locks. The needles are tailored to fit 17G × 200 mm RFA probes and include high-stiffness joints to interact with tough cirrhotic livers.

With these needles, path corrections of 20 mm for 100 mm deep targets were feasible with an absolute tip placement error of 1.0 ± 1.0 mm. Compared to the mean position error of 2.7 mm, which was the threshold accepted by a group of 125 interventional radiologists^4^, needles were placed sufficiently accurate for both straight and curved paths. After RFA probe insertion, the needle curvature systematically decreased and the mean placement error increased to 1.8 ± 1.1 mm. This likely resulted from a difference in flexural rigidity of the inserted steerable needle and RFA probe, causing a shift in the static equilibrium. Proper matching of the mechanical properties of the steerable needle with specific commercially available needles may therefore be of relevance. However, in case the tip is within, or sufficiently close to the targeted solid lesion, it is expected that structures displace together, reducing the clinical relevance of these shifts. For the water-filled cavities targeted in our study, this did not happen and displacements were measurable. Switching of instruments took on average 22 ± 9 s. This included removing the steerable stylet, inserting the RFA probe and re-adjusting the freehand ultrasound view.

The second validation step showed that similarly curved paths could be obtained when steering in cirrhotic human liver tissue. Path curvature variability within and between livers were found and expected, foremost because needles were inserted blind and without steering objective. In addition, needle deflections rely on tip-tissue force interactions^21^, and the mechanical properties of pathological livers deviated greatly. Currently, the number of included livers was too low to study relations between tissue conditions and steering responses.

The histological tissue damage observed can be related to various factors. To start, it should be realized that formalin fixation induces tissue-shrinkage^22^, resulting in observed hole sizes smaller than the cross-sectional area of introduced needles. In addition, tissue elasticity may cause partial gap-closure after needle retraction. Both needles had trocar tips with three cutting edges, the cutting lines of which are often distinguishable in the microscopic image (Fig. 4). In comparison, the study of Tsumura et al. used bevel-tipped needles that were continuously rotated^20^. The holes found had a more distinct round shape, which may be caused by the tissue-dilating nature of the tip type and insertion method. In turn, the three cutting lines created by a trocar tip may induce damage propagation through rupturing. To a minimal extent this was observed for all three conditions (i.e. cutting lines longer than the needle diameter). This typically happened along the outer lining of liver lobules. However, our data did not indicate that steering affected the occurrence of this response.

The histological analysis also indicated that straight path insertions caused more damage than steered insertions. However, we question this result and believe it may have been caused by an interacting effect of the amount of ink flushed through the puncture holes. It seems from Fig. 4 that this was on average more for the non-steered cases. The ink may have kept channels open. Still, the ink was useful to identify resection sites after slicing of the liver and helped increase the sheer number of data points obtained. In addition, the ink created sharp contours for image analysis. It was challenging to identify puncture holes in the highly textured cirrhotic liver slices. This was especially the case for the RFA probe, for which ink could not be used since the probe was not hollow. Upon histological examination, it was further found that the ink sometimes spread through microvasculature, causing false positives resection sites. This has resulted in uneven sample sizes, even though we started out with an equal number of needle insertions per condition. Overall, the histological analysis showed that tissue damage caused by the steerable needle corresponded to the needle size, both for straight and curved path insertions. Since needles of a similar size are used in daily practice, the damage was considered to be within clinically accepted limits.

Although needles are typically designed for single use, the prototypes presented were single-piece, in-house productions. All experimental runs were performed with the same two needles. The 72 insertions in PVA (v1), and 30 insertions in cirrhotic explant livers (v2) show that the proposed designs could easily withstand prolonged use.

Needles v1 and v2 have their similarities and differences. The stylets and compliant joints are made from the same material (nitinol, 1.30 mm in diameter). The v2 needle contains recesses at the joint in two perpendicular planes as is needed for omnidirectional tip steering, whereas the v1 needle contains recesses in one plane only. Still, users can produce the same tip angles with both needles. The v1 needle contains a nylon sheath, which increases its diameter. Due to the material choice, the impact on needle stiffness is limited. The tissue damage in livers is not yet tested for needle v1. Based on the presented work, for similarly curved paths and similarly stiff needles, we expect that the v1 needle causes larger puncture holes, proportional to the increase in needle cross-sectional area. We do not expect an increase in unplanned tissue damage propagation, e.g. tearing. Yet, this needs to be verified. In case of continued research with the v2 design, it will be important to quantify the value of out-of-plane steering and to test how well interventional radiologists can control this needle under 2D or 3D ultrasound.

The developed needles are presented and evaluated as manual devices for liver interventions, but the proposed steering mechanism can easily be incorporated in other medical applications or even robotic platforms. Removal of the levers in the needle hand pieces will expose rotational axes that can be coupled directly to one (v1) or two (v2) motors. Force measurements at the hub can be used to estimate needle bending forces^21^ for a controller, or to provide haptic or tactile feedback^23^. The used hydrophilic tubes reduce masking of tip-tissue interaction forces by friction along the needle shaft. Finally, the incorporated echogenic flexure joints^24^ can be used for real-time navigation of needles under ultrasound guidance.

In conclusion, two new types of echogenic steerable needles are presented with compliant joints that are both flexible enough to produce clinically relevant needle deflections and stiff enough to interact with tough cirrhotic liver structures. Needles are modular in design and optimized for 17G RFA probe placement. Needle steering has been evaluated by interventional radiologists, resulting in a mean placement error of 1.0 mm. A histological tissue damage evaluation in fresh explant tissues has demonstrated a potentially safe use of these needles for interventions in the liver.

## Methods

### Steering mechanism

**Figure 5:**
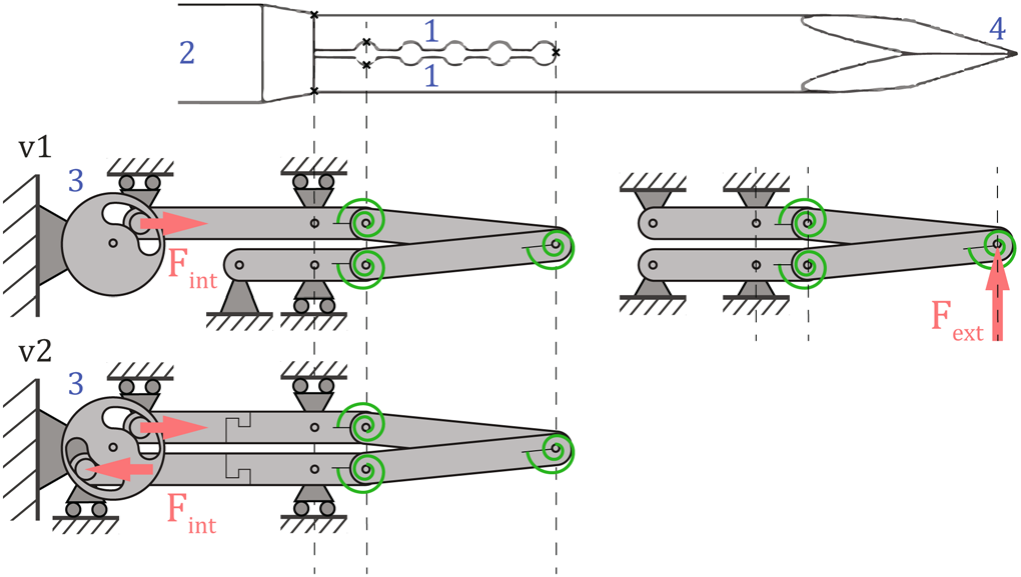
Pseudo-rigid body diagram of the steerable needles, showing two stylet members (1), as in needle v1. Cannula (2) guidance is illustrated with roller supports, and compliant segments are denoted by joints with torsional springs. The transmission wheel (3) enables relative stylet member translations and articulation of the trocar tip (4). Internal (F_int_) and external (F_ext_) forces are illustrated by arrows. The v2 needle contains four stylet members (circle quadrants), of which opposing pairs are linked by the transmission wheel. This wheel can rotate along the needle’s long axis to interlock with different opposing pairs. The rotation option is shown in Fig. 1, but not in this diagram to maintain the same 2D view.

Figure 5 and Table 1 present an overview of v1 and v2 needle characteristics, including diameters and flexural rigidity values. Used parts and materials include nitinol stylets, nitinol concentric tubes or cannulas, and PTFE-coated nylon sheaths (6F Flexor Ansel Guiding Sheath, Cook Medical, USA). The needle stylets are monolithic structures with recesses, introduced by electric discharge machining (EDM), that form compliant joint mechanisms in proximity to the tip^24^. Stylets are divided in two or four parallel running members, respectively. Relative longitudinal translations of stylet members result in joint actuations in their respective directions. Figure 5 shows a simplified pseudo-rigid body diagram of the needles. The *internal load* (F_int_) applied by the user by rotating a lever coupled to a transmission wheel, results in a stylet (member) translation and tip articulation. *External loads* (F_ext_), acting on the needle tip, will propagate along the needle stylet, but have a minute moment arm equal to the needle radius and will not be able to rotate the transmission wheel and lever, resulting at most in an elastic response of the needle. This is visualised by replacing roller supports by fixed supports in Fig. 5 (right), rendering a fixed truss structure with a known stiffness.

**Table 1:** Needle design specifications.

Notation	Property	v1	v2
*DOF*	Rotational degrees of freedom	1	2
*d*_*sheath*_	Sheath diameter (mm)	2.60	–
*d*_*cannula*_	Cannula diameter (mm)	1.65	1.80
*d*_*stylet*_	Stylet diameter (mm)	1.30	1.30
*EI*_*stylet*_	Stylet flexural rigidity (10^–3^ N m^2^)	6.8	6.1
*EI*_*joint*_	Joint flexural rigidity (10^–3^ N m^2^)	6.6	2.4
–	Hydrophilic sheath	✓	–
–	Echogenic tip	✓	✓

Stylet members of needle v2 run up to the proximal end of the needle, where they are fixed to the manual interface. In needle v1, one stylet member is fixed to the cannula distally, whereas the other continues as a single, solid stylet to the proximal end of the needle. This enables tip articulations by relative translations of the stylet and cannula. The distal fixture is a bayonet mount that preserves the ability to remove the stylet from the cannula.

### Needle design specifications

In absence of laterally protruding recesses in the compliant mechanism, the flexural rigidity (E∙I) of the v1 joint is similar to that of solid stylet regions. Flexural rigidity values in Table 1 were estimated with a constant Young’s Modulus of 50 GPa for the super-elastic nitinol components. For needle v2 a local reduction in flexural rigidity at the joint is seen, resulting from multiple adjacent material recesses for omnidirectional compliance. Yet, the remaining v2 joint rigidity approximates that of a 1 mm diameter solid nitinol stylet (2.5 × 10^–3^ N m^2^).

The manual interfaces include a lever and a long body that enables a pen grip. The lever is directly coupled to the transmission wheel. The wheel contains one (v1) or two (v2) slots with gradually varying radius, transforming lever rotations to translations of the stylet (v1) or of opposing stylet members (v2). The transmission wheel and lever of needle v2 can rotate along the longitudinal needle axis to lock in with a desired set of opposing stylet members. Adjacent stylet members can also be actuated simultaneously, providing eight principal steering directions.

### Needle trajectories

Automation of needle insertion and planning of trajectories are active research fields^25^. In previous work, we have used a bicycle kinematics-based model for articulated-tip needle steering and evaluated it with automated insertions^26^. Online parameter estimation enabled model adaptations to improve robustness in heterogeneous tissues. The needle facilitated similar-sized tip articulation angles as the currently presented manual needles.

In the current study, path planners were not implemented to minimize interference with the motor task. In the first evaluation, needles were inserted under ultrasound guidance. In the second evaluation, there were no targets to start with. Nevertheless, a constant curvature path approximation was assumed to fit needle trajectory data.

### Evaluation part I: intended users

The first evaluation experiment was performed by six interventional radiologists, experienced in RFA probe placement (3–9 years). The radiologists were asked to familiarize themselves with the needle by actuating it in air. Practice runs in phantom tissue were not allowed. During the experiment, partial needle retractions and path correction using the same tissue entry point were allowed. Radiologists had to control both the needle and ultrasound probe. The participant visible in Fig. 2A provided informed consent for the publication of this image.

### Evaluation part I: specimens

For the first part of the needle validation study, a polyvinyl alcohol (PVA) liver-mimicking phantom was made in accordance with de Jong et al*.*^27^. The solution contained 5 wt% PVA (Selvol PVOH 165, Sekisui Chemical Group NJ, USA), 38 wt% coolant (Carex, Burg Group, Netherlands) and 57 wt% water, and was subjected to three freeze–thaw cycles. Coolant was added to minimize volume expansion during PVA crystallization. Three targets were formed by placing cylindrical aluminium cores in the PVA solution, at a depth of 100 mm. Removal of the cores after PVA curing resulted in tubular water-filled cavities (targets) with a diameter of 10 mm.

### Evaluation part I: image acquisition

Steering tasks were captured with an ultrasound imaging device (iU22 xMatrix – DS, Philips, Netherlands), in conjunction with MeVisLab (2.7.1, MeVisLab, Germany). During post-processing, four curved and straight path captures appeared to contain corrupted files, leaving 32 datasets per condition. Frames containing the steerable needle and RFA probe in their final positions were selected for further analysis in Matlab (R2017b, MathWorks, USA).

### Evaluation part I: data processing

A graphical user interface (GUI) was built in Matlab for visual inspection of the ultrasound frames. The frames were subjected to a circle Hough Transform (CHT) to search targets. A two-stage method was used, based on the computation of radial histograms^28^, in which the target radius was allowed to deviate 1 mm from the expected value of 5 mm. A search sensitivity (0.95) and edge gradient threshold (0.1) were implemented for robust use in frames where targets were (partially) obscured by the needle or by reverberations. As the specimen contained three targets (Fig. 2B), the first CHT output was not necessarily the target that was steered to in that particular run. CHT outputs were manually examined in the GUI and the correct target centre position was stored in a mat-file.

A movable region of interest (ROI) was added to the GUI and placed over the needle at its distal end. The ROI was analysed by a Sobel convolution for edge detection (horizontal and vertical), and a Hough transform for line segment extraction. The Hough value threshold for inclusion was set to half the maximum encountered value. Line segments were merged if they were separated by 20 or less pixels that were associated with the same Hough transform bin. The longest retrieved line was stored in a mat-file and displayed in the GUI to represent the needle.

Needle positioning errors were defined by the orthogonal distance between a point (target centre, *t*) and a line (**p** = *p*_*2*_ – *p*_*1*_), where *p*_*1*_ and *p*_*2*_ are the needle segment end-points. Using **t** = *t* – *p*_*1*_*,* the scalar projection (*t*_*p*_) of *t* on **p**, is provided by a dot product (Eq. 1):

1 $${t}_{p}=\frac{\mathbf{t} \cdot \mathbf{p}}{\Vert \mathbf{p}\Vert }$$

Needle positioning errors (d_ε_) were defined by the Euclidean distance between *t* and *t*_*p*_:

2 $${\mathrm{d}}_{\upvarepsilon }=\Vert t-{t}_{p}\Vert$$

### Evaluation part I: statistical analysis

Positioning errors were registered (Eq. 2) after straight-path and curved-path insertions, for the steerable needle (before instrument switch) and RFA probe (after instrument switch). Data were summarized in boxplots and mean positioning errors were compared using a post-hoc Kruskal–Wallis test (α = 0.05), followed by Wilcoxon rank sum tests (α = 0.05). In addition, times required for needle insertion and instrument switching, as well as the number of path corrections, were registered.

### Evaluation part II: intended-use tissue types

Needles were inserted in cirrhotic liver explants of five patients (age 47–70, 4 males, 1 female). The found level of cirrhosis was estimated using a Child–Pugh score (CP range: A5-C11), and the severity of chronic liver disease with a Model for End-Stage Liver Disease score (MELD range: 7.5–33). RFA needles are typically used in similarly diseased liver tissues. Some of the livers contained tumours, but these were not targeted to minimize interference with pathological protocols.

All methods were performed in accordance with relevant guidelines and regulations. Informed consent was obtained and medical research ethics committee approval was obtained from Erasmus University MC, MEC-2019-0104.

### Evaluation part II: histological data collection

Before the insertions, the livers were photographed, measured and weighed, according to standard pathological examination protocols. Each liver was punctured through-and-through with the RFA probe (3×) and steerable needle (3× straight path, 3× curved path). After each puncture, a photograph was taken to evaluate needle paths (Powershot SX110 IS, Canon, Japan), using a macro digital imaging system (MacroPATH, Milestone Medical, Italy). All nine punctures were performed from the caudal-to-cranial direction. Upon steerable needle retraction, marking ink was flushed through the concentric tube to facilitate retrospective matching of puncture holes and test conditions. This was not done for the RFA probe, which had no hollow structure. Finally, the livers were sliced in the transversal plane, and the needles were thoroughly cleaned and rinsed with ethanol. By adhering to standard pathological slicing protocols (transversal planes), damage could be assessed in cross-sectional views. After slicing, areas with punctures were recognised and sampled for microscopical examination. Tissue samples were fixed in formaldehyde (4%) overnight and then embedded in paraffin. Formalin-fixed paraffin-embedded (FFPE) tissue blocks were cut in 5 µm thick slices and stained with Haematoxylin and Eosin (H&E) for histological interpretation. H&E stained slides were retrospectively reviewed using light microscopy and scanned (NanoZoomer S360, Hamamatsu Photonics, Japan) for digital data processing in Matlab. The resolution of stored microscopic images was 1088 px/mm.

### Evaluation part II: data processing

The macroscopic images were analysed by extrapolating the longitudinal axis of the needle hub and cannula before the entry point in tissue and measuring the depth and lateral offset at the needle exit point. This was done for both straight path and steered insertions. Exit point coordinates, path radii of curvature ($r$) and the median radius of curvature were computed. A median value was used, as needle deflections were not always found ($r=\infty$). Radii were obtained for needle exit coordinates $\left(x,y\right)$ using Eq. (3):

3 $$r= \frac{x \cdot \left({x}^{2}+{y}^{2}\right)}{2 \cdot x \cdot y}$$

The microscopy scans were analysed using a connected-components analysis (CCA). A binary image was constructed in which white background and inked areas were superimposed. A constant threshold value for the white background of 0.9 was used. Due to the H&E stain, the inked areas showed a strong gradient in the image’s red colour channel, which was subjected to Otsu's method for thresholding using a 256-bin image histogram^29^. An area opening step (Matlab, bwareaopen) was used to remove speckles with less than 1000 pixels (connectivity = 8). The CCA centroids were used to identify the cross-sectional surface area of interest.

### Evaluation part II: statistical analysis

Data were summarized with a mean and SD and cross-sectional areas were compared using a post-hoc Kruskal–Wallis test (α = 0.05), followed by Wilcoxon rank sum tests (α = 0.05).
